# Dietary supplements and prevention of preeclampsia

**DOI:** 10.1038/s41440-025-02144-9

**Published:** 2025-02-10

**Authors:** Takafumi Ushida, Sho Tano, Seiko Matsuo, Kazuya Fuma, Kenji Imai, Hiroaki Kajiyama, Tomomi Kotani

**Affiliations:** 1 Department of Obstetrics and Gynecology, Nagoya University Graduate School of Medicine, 65 Tsurumai-cho, Showa-ku, Nagoya, 466-8550 Japan; 2 Division of Reproduction and Perinatology, Center for Maternal-Neonatal Care, Nagoya University Hospital, 65 Tsurumai-cho, Showa-ku, Nagoya, 466-8550 Japan

**Keywords:** Calcium, Dietary supplement, Preeclampsia, Prevention, Vitamin D

## Abstract

Preeclampsia (PE) is a common pregnancy complication characterized by hypertension, proteinuria, and end-organ dysfunction. However, to date, no effective treatment has been established other than iatrogenic delivery, and the importance of prevention as an alternative approach to addressing PE has been emphasized. There is growing evidence on the effectiveness of pharmacological and non-pharmacological prophylaxis in preventing PE. In this review, we focused on dietary supplements as non-pharmacological prophylaxis for PE. Calcium is a well-documented supplement for the prevention of PE. Daily 500 mg calcium supplementation can roughly halve the risk of PE in settings where calcium intake is low, including in Japan. According to recent systematic reviews and network meta-analyses, current evidence on the efficacy of vitamin D supplementation is inconsistent. Although vitamin D is a candidate for the prevention of PE, future large-scale randomized control trials are necessary to draw definitive conclusions. We also reviewed other dietary supplements, including vitamins (vitamins A, B6, C, and E, folic acid, and multivitamins), minerals (magnesium, zinc, and iron), amino acids (l-arginine and l-carnitine), anti-oxidants (lycopene, resveratrol, and astaxanthin), and other agents (omega-3 fatty acids, coenzyme Q10, melatonin, and s-equol). In this study, we provide a comprehensive approach to help develop better preventive strategies and ultimately reduce the burden of PE.

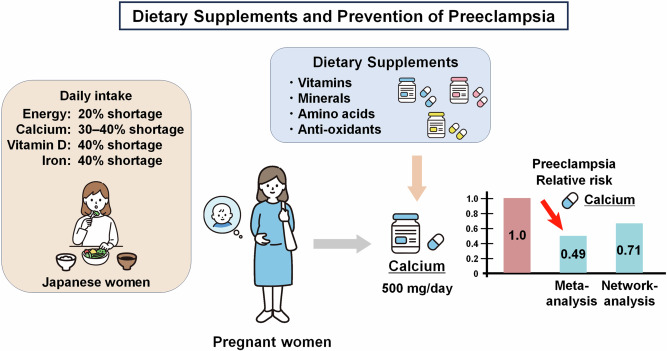

## Introduction

Preeclampsia (PE) is a pregnancy complication characterized by new-onset hypertension, proteinuria, and various end-organ dysfunctions, including the liver, brain, lung, and utero-placenta [1, 2]. Advanced maternal age, pre-pregnancy obesity, assisted reproductive technologies, and pre-existing conditions such as chronic hypertension and diabetes mellitus are among the primary risk factors associated with PE [1]. Although the global prevalence of PE has remained relatively stable in recent years, the reported trends vary according to geographic region [3, 4]. PE affects approximately 2.0% of pregnancies in Japan, posing significant risks to maternal and neonatal morbidity and mortality; additionally, it contributes to preterm births and fetal growth restrictions, affecting neonatal long-term adverse consequences [5–7]. Therefore, PE is a major global health issue that requires comprehensive strategies to improve maternal and neonatal health outcomes [8]. Currently, no effective treatment other than iatrogenic delivery has been established, despite constant efforts to develop new therapies and investigate the underlying mechanisms of PE. In these circumstances, the importance of prevention has been emphasized as an alternative approach to addressing PE.

Preventing PE is essential for global health from the perspective of better maternal and neonatal health outcomes, economic benefits, and reduced healthcare burden [8]. Effective programs for the prevention of PE, especially in low- and middle-income countries, can help bridge the global health gap and promote health equity by ensuring that all women have access to the necessary care. To date, the dedicated efforts of many researchers and healthcare professionals to investigate effective prophylactic treatments for PE have provided substantial evidence [1, 2, 9]. There are two main approaches to PE prevention: the first is lifestyle modifications and improvement of modifiable risk factors, and the second is pharmacological (e.g., low-dose aspirin) and non-pharmacological prophylaxes, including dietary supplements.

Dietary supplements, also known as nutritional or food supplements, are products intended to add nutritional value to the diet [10, 11]. Supplements can provide nutrients, either extracted from food sources or synthetically, individually or in combination, to increase nutrient intake. Supplements play a significant role in disease prevention by filling nutritional gaps, optimizing physiological functions, and improving innate immune responses. Supplements are particularly important during pregnancy from the standpoint of satisfying nutritional gaps attributable to physiologically increased nutritional needs during pregnancy, preventing birth defects, supporting fetal development, improving maternal health, and reducing pregnancy complications [12].

The hypothesis that PE can be prevented through nutritional supplementation originates from the understanding that nutritional status significantly influences perinatal outcomes [12]. This hypothesis is based on the idea that PE involves endothelial dysfunction, oxidative stress, and placental insufficiency, therefore dietary supplements, particularly those with anti-oxidant properties, may help counteract these issues [13]. Furthermore, epidemiological observations have suggested that several nutrients and dietary factors, including calcium and vitamin D, are involved in the risk of PE [14, 15]. Thus, the basis for the hypothesis about dietary supplements as a preventive measure for PE is the underlying pathophysiology of the disease, observational data suggesting an association between nutrient intake and reduced risk, and clinical data indicating potential benefits of supplementation.

The growing global emphasis on preconception care reflects its critical role in optimizing maternal and child health outcomes. In Japan, the approval of the Basic Policy on Child and Maternal Health and Child Development by the Cabinet in 2021 underscored the importance of this concept, with a particular focus on preventing and mitigating the risks of pregnancy complications, including PE [16]. Considering the current nutritional status of Japanese women, we reviewed systematic reviews and meta-analyses to evaluate the efficacy of various dietary supplements, including vitamins (vitamins A, B6, C, D, and E, folic acid, and multivitamins), minerals (calcium, magnesium, zinc, and iron), amino acids (l-arginine and l-carnitine), anti-oxidants (lycopene, resveratrol, and astaxanthin), and other agents (omega-3 fatty acid, coenzyme Q10, melatonin, and s-equol), in preventing PE during pregnancy. By providing a comprehensive perspective based on the concept of preconception care, we aim to inform the development of more effective preventive strategies and contribute to reducing the burden of PE in Japan.

## Dietary supplements in Japan

Dietary supplements are products intended to supplement a diet and provide nutrients that may not be consumed in sufficient quantities through food [12]. These include vitamins, minerals, amino acids, enzymes, and anti-oxidants. In many countries, supplements are regulated as food (e.g., non-pharmacological agents) rather than as drugs. Dietary supplements differ from pharmacological agents that are generally regulated as medications and require clinical trials to demonstrate their efficacy and safety for specific preventive uses. In Japan, dietary supplements are classified as “health foods” and further divided into Foods with Health Claims and other health foods [17]. Foods with Health Claims are legally permitted to display specific health functions, whereas other health foods are included in general food categories and cannot promote such functions. Foods with Health Claims include Foods for Specified Health Uses (FOSHU), Foods with Nutrient Function Claims (FNFC), and Foods with Function Claims (FFC). FOSHU undergoes government approval, whereas FNFC and FFC rely on self-certification or manufacturer responsibility. Unlike pharmaceuticals, health foods are intended for healthy individuals, not patients, and lack uniform quality. Regulatory measures, such as the Food Sanitation Act and Good Manufacturing Practices, ensure safety, but challenges persist [17]. The issues include inconsistent quality, underreported adverse events, and consumer misconceptions, such as equating supplements with medications. Strengthening reporting systems, improving public awareness, and stricter quality control are necessary to address these concerns and ensure consumer safety.

## Indices for dietary reference intakes

**Fig. 1:**
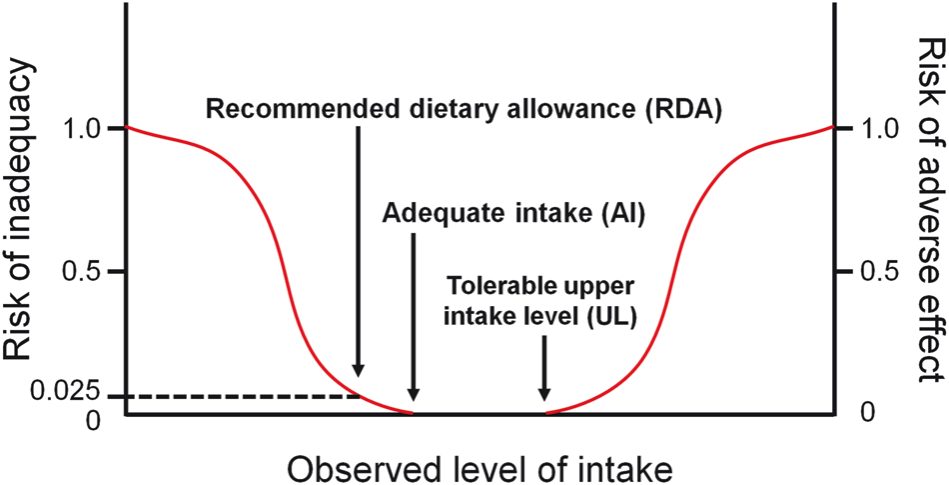
Dietary reference intakes. Schematic diagram illustrating the risks of nutrient inadequacy and adverse effects

According to the dietary reference values and daily intake values for the Japanese population provided by the Ministry of Health, Labor, and Welfare on 2020 and the National Health and Nutrition Survey in Japan on 2019, the recommended dietary allowance (or adequate intake), tolerable upper intake level, and average daily intake value of each nutrient are shown in the Tables 1, 2 [18, 19]. The recommended dietary allowance refers to the level of average daily dietary intake sufficient to meet the nutrient requirements of nearly all (97–98%) healthy individuals in a specific age and sex group. Adequate intake is the recommended value when there is insufficient scientific evidence to establish a recommended dietary allowance. Adequate intake is established at a level assumed to ensure nutritional adequacy. Tolerable upper intake level is the maximum daily intake of a nutrient that is unlikely to cause adverse health effects in most individuals of a specific age and sex group. The tolerable upper intake level is not intended to be the recommended level of intake but rather a threshold to avoid exceeding. Figure 1 shows a graphic representation of the recommended indices for reference dietary intake. The average daily intake was estimated by investigating the food intake based on the National Health and Nutrition Survey [19]. Tentative Dietary Goal for Preventing Lifestyle-Related Diseases (DG) represents the target intake of nutrients (e.g., sodium, saturated fatty acids, and dietary fiber) aimed at maintaining health and contributing to the prevention of lifestyle-related diseases by keeping intake within a certain range [18]. The estimated shortage (%) is given by the formula [1 − (average daily intake/recommended dietary allowance or adequate intake or DG)] × 100.

**Table 1:** Daily reference value for each nutrient based on the dietary reference intake for Japanese published in 2020

	18–29 years		30–49 years		During pregnancy			During lactation
	RDA/AI/DG	UL	RDA/AI/DG	UL	1st trimester	2nd trimester	3rd trimester	
Energy (kcal)*1	2000		2050		+50	+250	+450	+350
Protein (g)	50		50		+0	+5	+25	+20
Dietary fats (% energy)	20–30		20–30		+0			+0
Carbohydrates (% energy)	50–65		50–65		+0			+0
Dietary fiber (g)	≥18		≥18		+0			+0
Vitamin A (μg RAE)	650	2700	700	2700	+0	+0	+80	+450
Vitamin B6 (mg)	1.1	45	1.1	45	+0.2			+0.3
Vitamin C (mg)	100		100		+10			+45
Vitamin D (μg)	8.5	100	8.5	100	+0			+0
Vitamin E (mg)	5.0	650	5.5	700	6.5			7.0
Folate (μg)	240	900	240	1000	+240			+100
Sodium (g)*2	<6.5		<6.5		+0			+0
Calcium (mg)	650	2500	650	2500	+0			+0
Magnesium (mg)	270		290		+40			+0
Zinc (mg)	8	35	8	35	+2			+4
Iron (mg)	10.5	40	10.5	40	9.0	16	16	9.0
Omega-3 fatty acid (g)	1.6		1.6		+0			+0.2

RDA recommended dietary allowance, AI adequate intake, DG tentative dietary goal for preventing lifestyle related diseases, UL tolerable upper intake level, μg RAE, μg retinol activity equivalents. *1 estimated energy requirement for women with physical activity level II. *2 sodium chloride equivalent

**Table 2:** Daily intake value and shortage for each nutrient based on the National Health and Nutrition Survey in Japan published in 2019

	20–29 years		30–39 years		40–49 years		During pregnancy*1	Shortage*2
	mean ± SD	median	mean ± SD	median	mean ± SD	median	median (IQR)	%
Energy (kcal)	1600 ± 445	1567	1673 ± 475	1642	1729 ± 457	1714	1620 (1311–2015)	20.0
Protein (g)	61.1 ± 18.4	60.6	61.6 ± 20.3	59.9	65.9 ± 21.0	64	13.5 (12.2–14.8)	-
Dietary fats (% energy)	30.9 ± 8.2	29.9	31.1 ± 8.1	30.3	30.3 ± 7.4	30.6	29.8 (25.6–34.1)	-
Carbohydrates (% energy)	53.6 ± 8.9	54.2	54.0 ± 9.4	54.7	54.4 ± 8.4	54.5	55.3 (50.2–60.3)	6.8
Dietary fiber (g)	14.6 ± 5.7	14	15.9 ± 6.3	15.2	16.0 ± 5.5	15.5	9.6 (7.1–12.9)	18.9
Vitamin A (μg RAE)	447 ± 878	292	409 ± 288	337	458 ± 644	341	404 (265–631)	31.2
Vitamin B6 (mg)	0.91 ± 0.38	0.87	0.96 ± 0.41	0.91	1.01 ± 0.41	0.95	0.93 (0.71–1.22)	17.3
Vitamin C (mg)	62 ± 48	51	65 ± 44	56	74 ± 50	64	74 (48–109)	38.0
Vitamin D (μg)	4.6 ± 5.9	2.3	4.9 ± 8.1	2.3	5.3 ± 6.7	2.4	3.9 (2.3–6.1)	45.9
Vitamin E (mg)	5.4 ± 2.8	5	6.1 ± 3.2	5.5	6 ± 2.8	5.6	5.7 (4.2–7.7)	-
Folate (μg)	226 ± 129	209	233 ± 111	215	247 ± 108	230	229 (166–312)	5.8
Sodium (g)*3	8.3 ± 3.1	8.1	8.5 ± 3.3	8.5	8.9 ± 3.2	8.5	–	-
Calcium (mg)	408 ± 210	393	406 ± 231	364	441 ± 236	382	–	37.2
Magnesium (mg)	192 ± 72	186	205 ± 79	193	219 ± 74	214	–	28.9
Zinc (mg)	7.3 ± 2.7	7.1	7.3 ± 2.6	6.9	7.8 ± 2.9	7.4	–	8.8
Iron (mg)	6.2 ± 2.5	5.9	6.4 ± 2.5	6.1	6.7 ± 2.4	6.3	–	41.0
Omega-3 fatty acid (g)	1.82 ± 1.2	1.5	2.01 ± 1.67	1.6	2.05 ± 1.45	1.66	–	–

SD standard deviation, IQR interquartile range, μg RAE μg retinol activity equivalents. *1 Median daily intake value during pregnancy was based on the report by Eshak et al. [22]. *2 The estimated shortage (%) in a 20–29-year-old is given by the formula [1 − (average daily intake/recommended dietary allowance or adequate intake or DG in 18–29-year-old)] × 100. *3 sodium chloride equivalent

## Diets of women of reproductive age and pregnant women in Japan

The diets of Japanese women of reproductive age, as well as pregnant women, are characterized by common issues such as inadequate energy intake and specific nutrient deficiencies [20, 21]. For women in their 20 s and 40 s with level II physical activity, the average daily energy intake is approximately 1600–1700 kcal, which is significantly short of the recommended 2000–2050 kcal [18, 19]. The median daily intake values during pregnancy in Japan (Table 2) were based on the report by Eshak et al. [22]. Pregnant women also consume approximately 1600–1700 kcal per day, which is significantly below the recommended range of 2050–2500 kcal [22, 23]. According to the 2020 National Health and Nutrition Survey, the average daily salt intake (sodium chloride equivalent) for women in their 20 s and 30 s is approximately 8.3–8.9 g (Table 2), and pregnant women consume a similar or lower amount. This far exceeds both the WHO recommendation of <5 g per day and Japanese Ministry of Health, Labor and Welfare’s limit of <6.5 g per day [18, 24]. Key nutrients, such as calcium, iron, and vitamin D, are also consumed at inadequate levels in women aged 20–29 years, with an estimated shortage of 37–46% below recommended values (Table 2). These inadequacies can increase the risk of osteoporosis, anemia, and impaired fetal development. Furthermore, not only nutrient deficiencies but also general nutritional imbalances can contribute to increase the risk of PE and other pregnancy-related complications. Therefore, comprehensive dietary guidance and appropriate supplementation are essential to promote maternal and fetal health.

## Dietary supplements

### Vitamins

#### Vitamin A

Although vitamin A plays an important role during pregnancy, contributing to fetal development, including that of the heart, kidneys, lungs, eyes, and skeleton [25], excessive intake of vitamin A increases fetal morphological abnormalities because vitamin A, a fat-soluble vitamin, tends to accumulate in the body. Vitamin A has anti-oxidant properties primarily owing to the action of carotenoids, particularly beta-carotene, which is a precursor of vitamin A. Liu et al. demonstrated a negative correlation between vitamin A intake, evaluated using the semi-quantitative Food Frequency Questionnaire [26], and the risk of PE (adjusted odds ratio [OR]: 0.62, 95% confidence interval [CI]: 0.40–0.96, *p* trend = 0.02) [27]. However, to date, no randomized controlled trials (RCTs) have evaluated the efficacy of vitamin A supplementation in the prevention of PE. Currently, routine vitamin A supplementation is not recommended to prevent PE.

#### Vitamin B6

Vitamin B6, also known as pyridoxine, is important for the development of the fetal brain and nervous system [28]. Vitamin B6 aids in the biosynthesis of neurotransmitters and contributes to the synthesis of sphingolipids, which are key components of the myelin sheath that support neuroplasticity, brain communication, and function [29]. A review by Cochrane in 2015 did not show a significant association between antenatal oral vitamin B6 intake and prevention of PE (risk ratio [RR]: 1.71, 0.85–3.45) (2 studies, *n* = 1,197, low quality evidence) [28]. These data do not support routine vitamin B6 supplementation for the prevention of PE.

#### Vitamin C

Vitamin C, also known as ascorbic acid, plays an important role in the synthesis of collagen (a vital component of connective tissues), anti-oxidant protection, immune system support, tissue repair, and iron absorption [30]. According to a Cochrane review in 2015, there is no clear evidence that vitamin C, whether taken alone or in combination with other vitamins, reduces the risk of PE (RR: 0.92, 0.80–1.05) (16 studies, *n* = 21,956, high-quality evidence) [31]. Among the 16 studies evaluated, a multicenter RCT published in 2006 for nulliparous women in the second trimester showed that daily supplementation with 1000 mg of vitamin C and 400 IU of vitamin E did not reduce the risk of PE (RR: 1.20, 0.82–1.75) [32]. Existing evidence does not currently support the routine use of vitamin C supplements for the prevention of PE.

#### Vitamin D

Vitamin D plays an important role for both mothers and developing fetuses in maintaining proper levels of calcium and phosphorus through absorption in the intestine and liver, thus, promoting bone health [33]. Recently, vitamin D has also been reported to enhance immunity, improve endothelial function, and prevent lifestyle-related diseases and depression [34, 35]. Nema et al. found that vitamin D supplementation enhances angiogenic factors in PE: vitamin D increases the levels of vascular endothelial growth factor and placental growth factor [36]. Furthermore, vitamin D supplementation reduces blood pressure and proteinuria in several types of animal models of PE by improving placental vascular endothelial function and normalizing angiogenic imbalance [37–39]. Based on these clinical and preclinical studies, vitamin D supplementation has been hypothesized to contribute to the prevention of PE.

Vitamin D is a fat-soluble vitamin that can accumulate in the body with excessive intake, raising concerns about possible adverse effects on the fetus. However, no clear evidence of a direct association between vitamin D overconsumption and fetal malformations is available [40, 41]. Generally, a daily intake of up to 100 μg (=4,000 IU) is considered safe during pregnancy [41]. Given that the recommended daily intake of vitamin D during pregnancy ranges from 15 to 100 μg, the risk of adverse health effects on pregnant women or their fetuses due to the accumulation of vitamin D is considered minimal. In Japan, the average daily vitamin D intake for women aged 20–49 years is 4.6–5.3 μg, which is approximately 45% lower than the recommended intake of 8.5 μg (Tables 1, 2) [18, 19].

A meta-analysis of RCTs in 2020 (27 studies, *n* = 4777) showed a significant reduction in PE (OR: 0.37, 0.26–0.52) [42]. This suggested that starting vitamin D supplementation by 20 weeks of gestation is optimal, and higher doses of vitamin D are associated with a greater reduction in the incidence of PE. However, the findings on the optimal dose of vitamin D remain inconsistent. Irwinda et al. showed that a lower dose of vitamin D supplementation (≤2000 IU [ = 50 μg]/day) significantly reduced the risk of PE, with no significant difference compared to that with a higher dose supplementation (>2000 IU/day) [43]. Similarly, Palacios et al. suggested that a lower vitamin D dose of 600 IU ( = 15 μg), which corresponds to the recommended dietary allowance, may be sufficient, as it yields a PE preventive effect comparable to that with higher doses [41]. A Cochrane review in 2019 also showed a prophylactic effect of vitamin D supplementation on PE (RR: 0.48, 0.30–0.79) (4 studies, *n* = 499, moderate quality evidence) [44]. Additionally, a recent network meta-analysis of 130 RCTs (*n* = 1,122,916) related to PE prophylaxis published in 2022 showed that low-molecular weight heparin (RR: 0.60, 0.42–0.87), aspirin (RR: 0.79, 0.72–0.86), vitamin D (RR: 0.65, 0.45–0.95), calcium (RR: 0.71, 0.62–0.82), and exercise (RR: 0.68, 0.50–0.92) are effective in preventing PE [45].

However, more recent evidence has questioned the effectiveness of vitamin D supplementation for preventing PE. Three systematic reviews, including a Cochrane review published in 2024, found no clear benefit of vitamin D supplementation to reduce the risk of PE [46–48]. Although vitamin D has been considered a candidate for the prevention of PE, the current findings are inconsistent. The available evidence does not provide a definitive answer about whether vitamin D supplementation can effectively prevent PE; thus, its role remains uncertain. To better understand its potential benefits, future large-scale RCTs are necessary.

#### Vitamin E

Vitamin E serves as an anti-oxidant that protects cells from damage by free radicals [49]. Furthermore, vitamin E is associated with immune system support, cellular function and development, red blood cell formation, and skin health [50]. According to a Cochrane review in 2015, compared to placebo, vitamin E combined with other supplements during pregnancy did not show a significant reduction in the risk of PE (RR: 0.91, 0.79–1.06) (14 studies, *n* = 20,878, moderate quality evidence) [51]. This result is comparable to that of women at high risk of PE [52, 53]. By contrast, vitamin C and E supplementation may have an adverse effect on gestational hypertension (RR: 1.11, 1.05–1.17) (7 studies, *n* = 19,003), according to a systematic review and meta-analysis of RCTs published in 2011 [54]. Existing evidence does not recommend the routine use of vitamin E supplements for prevention of PE.

#### Folic acid

Folate and folic acid, two forms of vitamin B9, are available in two distinct forms: natural folate and synthetic folic acid. Natural folate, found in foods such as leafy greens, fruits, and legumes, is less stable and more prone to degradation by heat and light during cooking and storage. By contrast, folic acid, the synthetic form commonly used in supplements and fortified foods, is more stable and resistant to environmental factors. However, folate is less bioavailable than folic acid with a median relative bioavailability of 65% (range: 44–80%) [55]. Natural folate is predominantly converted to its bioactive form, 5-methyltetrahydrofolate (5-MTHF), in the digestive system, allowing it to be readily used by the body. Conversely, folic acid undergoes a slower and less efficient conversion process. After absorption into the digestive tract, folic acid must be metabolized in the liver into 5-MTHF before it becomes biologically active [55]. Folate/folic acid is crucial during pregnancy in preventing neural tube defects (e.g., spina bifida and anencephaly), supporting DNA synthesis and cell growth, and forming red blood cells [56]. Additionally, folate/folic acid plays a protective role in the prevention of trophoblast apoptosis linked to homocysteine, which may improve trophoblast invasion and placental development [57].

A meta-analysis in 2018 showed a significant reduction in PE by folic acid supplementation (RR: 0.69, 0.58–0.83) (12 studies, *n* = 304,135) [58]. However, previous studies included in the meta-analysis were not uniform in their choice of supplement type and dose, with a significant selection bias and heterogeneity. Additionally, a recent meta-analysis in 2024 demonstrated that folic acid supplementation during pregnancy was not associated with a decreased risk of PE, based on cohort studies (RR: 0.57 0.31–1.05) (5 studies, *n* = 208,270) and RCTs (RR: 1.41, 0.80–2.50) (2 studies, *n* = 2729, low quality evidence) [59]. Therefore, investigations of the association between folic acid supplementation and PE preventive effects have yielded inconsistent conclusions. Currently, it is not recommended to prescribe folic acid for the prevention of PE.

#### Multivitamins

Multivitamins, which contain a combination of various vitamins and minerals, are designed to bridge nutritional gaps and ensure individual health. Since multivitamins contain anti-oxidants, daily multivitamin supplementation during pregnancy is speculated to reduce the risk of PE.

Although two RCTs were identified according to a systematic review in 2022, they were not compatible with the meta-analysis owing to clinical heterogeneity [60]. The two RCTs showed a significantly decreased risk of PE in multivitamin users; however, the sample sizes were small (*n* = 90 and 60) [61, 62]. Observational studies using a random effects model did not show a significant reduction in risk by multivitamin supplementation (RR: 0.85, 0.69–1.03) (4 studies, *n* = 33,206, low quality evidence) [60]. Therefore, the accumulated evidence does not support the routine use of multivitamin supplementation to prevent PE.

### Minerals

#### Calcium

Calcium plays an important role in the regulation of vascular smooth muscle and vascular endothelial function and contributes to normal blood pressure regulation [63, 64]. Adequate calcium level helps maintain proper parathyroid hormone function and indirectly influences blood pressure regulation. Calcium supplementation prevents endothelial cell activation induced by various placenta-derived factors, involving the nitric oxide synthase pathway and anti-inflammatory mechanisms [65, 66]. In addition, calcium supplementation improves uteroplacental and fetoplacental blood flow [67]. Since 2011, the World Health Organization (WHO) has recommended calcium supplementation to prevent PE in pregnant women with low calcium intake [68]. A recent meta-analysis in 2022 showed that calcium supplementation significantly reduced the risk of developing PE (RR: 0.49, 0.39–0.61) (30 studies, *n* = 20,445) [69]. This meta-analysis includes a prospective study conducted in Japan, reported by Ito et al. in 1994 [70]. The inclusion of Japanese data in the meta-analysis is highly significant for assessing the applicability of the results to the Japanese population. The risk-reducing effect for PE was similar between low-dose (<1 g/day) and high-dose (≥1 g/day) calcium supplementation and between high- and low-risk patients. In particular, the risk-reducing effect of calcium supplementation was observed only in the population with low calcium intake (RR: 0.45, 0.35–0.58) and was not significant in populations with adequate calcium intake (RR: 0.62, 0.37–1.06). To date, the evidence for calcium supplementation is of high quality; therefore, calcium supplementation is recommended during pregnancy in settings where calcium intake is low (e.g., <800 mg/day) [71].

According to the 2019 National Health and Nutrition Survey by the Ministry of Health, Labor, and Welfare in Japan, the average daily calcium intake in Japan is 400–450 mg for women aged 20–49 years, which is approximately 30–40% short of the recommended value of 650 mg (Tables 1 and 2) [18, 19]. In Japan, the dairy calcium intake is clearly inadequate compared to that in other countries: United States (950 mg), Latin American countries (622 mg), Asia-Pacific countries (653 mg), and African countries (566 mg) [72, 73]. Therefore, calcium supplementation is expected to be effective in preventing PE in Japan, one of the countries with low calcium intake.

Although the WHO currently recommends a calcium supplementation dose of 1500–2000 mg/day, evidence reported by Dwarkanath et al. provides an opportunity to address this issue [74]. The authors conducted a large RCT that enrolled >20,000 pregnant women in two countries with low calcium intake (India and Tanzania) and found that low-dose supplementation (500 mg/day) was not inferior to high-dose supplementation (1500 mg/day) in preventing PE.

By contrast, excessive calcium intake is not entirely free of concern: although the conclusions of the recent meta-analyses regarding the association between excessive calcium intake and cardiovascular disease risk differs, there are several reports indicating that calcium supplementation (>1000 mg/day) increases the risk of cardiovascular side effects [75, 76]. Considering that the average daily calcium intake of women of reproductive age in Japan is approximately 500 mg, and the tolerable upper limit of calcium intake established by the Ministry of Health, Labor, and Welfare is 2500 mg/day, calcium supplementation of 1000–1500 mg is generally safe. Considering the above, low-dose supplementation (500 mg) is reasonable from the standpoint of cost and effort of high-dose supplementation.

Although there is insufficient evidence on the timing of prophylactic supplementation, it is recommended to start as soon as possible after pregnancy confirmation [77]. The benefits of calcium supplementation before or at the beginning of pregnancy are not well established [78]. Regarding the type of calcium supplementation, calcium citrate, calcium carbonate, and calcium gluconate are available options, but calcium carbonate is considered the most cost-effective [79]. Calcium carbonate is inexpensive and more bioavailable than calcium gluconate; however, its absorption may be reduced if consumed between meals. The bioavailability of calcium citrate is not affected by diet; however, its high cost and the nearly 50% lower calcium content by weight are notable disadvantages [79]. Calcium is typically consumed as a dietary supplement. From the insurance coverage perspective in Japan, L-aspartate calcium can be prescribed because the indications on the drug information for L-aspartate calcium state “calcium supplementation during pregnancy and lactation.” Thus, low-dose calcium supplementation (500 mg/day) is recommended during pregnancy in regions with low calcium intake, including in Japan

#### Magnesium

As a coenzyme, magnesium assists in >600 enzymatic reactions, including energy metabolism, protein synthesis, gene expression, and nerve transmission [80]. Magnesium also regulates muscle contraction by antagonizing calcium, resulting in dilation of blood vessels and lower blood pressure [81]. Considering that magnesium sulfate administered to women with PE can prevent eclampsia and slow its progression, magnesium-containing agents may have a preventive effect on the development of PE [1]. A meta-analysis of RCTs in 2022 showed that oral magnesium supplementation during pregnancy significantly reduced the risk of PE (RR: 0.76, 0.59–0.98) (7 studies, *n* = 2,653, moderate quality evidence) [82]. However, a subgroup analysis did not demonstrate a significant reduction in the risk of PE in healthy pregnant women (RR: 0.91, 0.67–1.25) [82]. Therefore, current evidence does not support the routine use of oral magnesium supplementation for prevention of PE.

#### Zinc

Zinc is an essential trace element that cannot be produced in the body. It is found in approximately 2–4 g of the body and is abundant in the teeth, bones, muscles, liver, and kidneys. Zinc is a cofactor for >300 enzymes involved in various biochemical processes, including DNA synthesis, RNA transcription, cell division, and protein synthesis [83]. Although low zinc concentrations have been associated with the development of PE in some reports [84, 85], a meta-analysis found that zinc supplementation did not contribute to the prevention of PE (2 studies, *n* = 4201) [86]. A Cochrane review in 2021 also demonstrated that zinc supplementation during pregnancy had no significant effect on PE prevention (RR: 0.93, 0.62–1.42) (6 studies, *n* = 2568) [87] Therefore, zinc supplementation is not recommended for PE prevention.

#### Iron

Iron plays a crucial role during pregnancy because it is involved in several physiological processes essential for maternal and fetal health. Increased blood volume during pregnancy requires higher iron levels to sustain adequate hemoglobin production for oxygen transport to both the mother and the developing fetus [88]. In addition, iron is vital for the development of the fetal brain and muscle and contributes to optimal growth and developmental outcomes. A Cochrane review in 2015 did not show a significant effect of iron supplementation on prevention (RR: 1.63, 0.87–3.07) (4 studies, *n* = 1704) [89]. Additionally, iron supplementation in pregnant women without anemia has not shown any benefits [90, 91]. However, a retrospective study showed that iron supplementation reduced the odds of PE (adjusted OR: 0.75, 0.61–0.91) among pregnant women with anemia [92]. By contrast, women with untreated anemia had significantly increased odds of PE (adjusted OR: 1.44, 1.25–1.67), suggesting the effectiveness of iron supplementation for women with anemia [92]. However, existing evidence does not support the routine use of iron supplements for the prevention of PE in all pregnant women.

### Amino acids

#### L-arginine

L-arginine is an amino acid that serves as a precursor to nitric oxide, a molecule that helps dilate blood vessels, regulate blood pressure, and support immune function [93]. During pregnancy, l-arginine contributes to increasing nitric oxide production, which helps improve blood flow, thereby supporting placental vascular development and fetal growth [94]. Although a meta-analysis in 2007 showed no significant effect of l-arginine on PE prevention (RR: 0.83 [0.49–1.41]) [95], a recent meta-analysis in 2021 showed a preventive effect on PE reduction (RR: 0.38 0.25–0.58) (2 studies, *n* = 524) in women in the second and third trimesters, including women at high risk for PE [96]. This meta-analysis also showed that l-arginine had a therapeutic effect on lowering systolic blood pressure (−2.47 mmHg, −4.53 to −0.42) (3 studies, *n* = 214). Another meta-analysis conducted in 2023 reported a similar therapeutic effect in women with hypertensive disorders of pregnancy [97]. However, large-scale RCTs are required to determine the effects of l-arginine on PE prevention.

#### L-carnitine

L-carnitine is crucial for the transport of long-chain fatty acids into the mitochondria, where fatty acids are oxidized for energy production [98]. L-carnitine also plays a role in metabolism and supports cardiovascular health and exercise performance. During pregnancy, l-carnitine supports maternal energy levels by facilitating fatty acid oxidation and contributing to the proper functioning of mitochondrial processes, which may be important for fetal growth and fetoplacental unit function [99]. Increased plasma l-carnitine concentrations have been observed in women with PE and in PE animal models [100, 101]. To date, no studies have examined the effects of l-carnitine supplementation on the prevention of PE.

### Anti-oxidants

#### Lycopene

Lycopene is a carotenoid with strong anti-oxidant properties. Lycopene helps neutralize free radicals, thereby reducing oxidative stress and inflammation, and is associated with various health benefits, including cardiovascular protection [102]. During pregnancy, lycopene plays an important role in the regulation of anti-oxidant mechanisms, anti-inflammatory effects, and lipid metabolism, which could support modulation of placental health and fetal development [103, 104]. Two RCTs produced differing results on the efficacy of lycopene in preventing PE [105, 106]. One study found that lycopene reduced the development of PE (8.6% with lycopene vs 17.7% with placebo; *p* = 0.04) [105]. However, lycopene did not prevent PE in healthy primiparous women (18.2% with lycopene vs 18.3% with placebo, *p* = 0.99) [106]. Currently, there is a lack of sufficient data to draw conclusions about the preventive effects of lycopene.

#### Resveratrol

Resveratrol is a polyphenolic compound found in red wine and certain plants and is known for its anti-oxidant and anti-inflammatory properties. Resveratrol has a variety of health-promoting effects, such as cardiac remodeling, reduction of oxidative stress, neurogenesis, and anti-aging [107, 108]. In pregnancy, resveratrol supplementation in animal models has been reported to have potential beneficial effects in adverse pregnancy complications, such as PE and gestational diabetes mellitus [109–111]. One study on pregnant women with PE showed that the time needed to control blood pressure was significantly reduced in the resveratrol plus nifedipine-treated women compared with that in women treated with placebo plus nifedipine [112]. However, to date, no studies have examined the effects of resveratrol supplementation on the prevention of PE.

#### Astaxanthin

Astaxanthin is a carotenoid with stronger anti-oxidant activity than Coenzyme Q10, vitamin C, and vitamin E, and has various potential effects against cardiovascular disease, cancer, and diabetes mellitus [113]. Based on its multifaceted biological activities, astaxanthin supplementation is expected to effectively prevent PE. Xuan et al. demonstrated the therapeutic effects of astaxanthin on pre-eclamptic symptoms, oxidative stress, and placental inflammatory damage in a rat model of PE [114]. However, to date, no studies have evaluated the effects of astaxanthin supplementation on PE prevention.

### Other agents

#### Omega-3 fatty acids

Omega-3 fatty acids, including eicosapentaenoic and docosahexaenoic acids, are essential fatty acids with various biological effects that support cardiovascular health, reduce inflammation, and promote brain function [115]. During pregnancy, omega-3 fatty acids are important components of the fetal brain and retina and are crucial for the neurodevelopment of the fetal and infant central nervous system [116]. In 2018, a Cochrane review demonstrated that omega-3 fatty acid supplementation during pregnancy tended to reduce the risk of PE compared with placebo or no intervention (RR: 0.84, 0.69–1.01) (20 studies, *n* = 8306, low quality evidence); however, the result was not significant [117]. In 2020, a meta-analysis showed a significant protective role against the risk of PE (RR: 0.82, 0.70–0.97) (14 studies, *n* = 10,806) [118]. Similarly, a meta-analysis of RCTs in 2022 showed a significantly reduced risk of PE (RR: 0.75, 0.57–0.98) (8 studies, *n* = 8741, high-quality evidence) [119]. These results suggest that omega-3 fatty acid supplementation during pregnancy may exert favorable effects against PE; however, sufficient evidence is not available to draw definitive conclusions to recommend supplementation for all pregnant women. Therefore, further large-scale RCTs are required to determine the effects of omega-3 fatty acids on the prevention of PE.

#### Coenzyme Q10

Coenzyme Q10, also known as ubiquinone, is a naturally occurring anti-oxidant found in mitochondria. Coenzyme Q10 plays a crucial role in energy production through the electron transport chain, which is essential for cellular respiration and synthesis of adenosine triphosphate [120]. Coenzyme Q10 has gained attention for its positive effects on various disorders, such as cardiovascular disease, diabetes mellitus, and neurodegenerative diseases [121, 122]. There is a significant decrease in plasma levels of coenzyme Q10 in women with PE compared to normal pregnant women [123]. Therefore, it is speculated that coenzyme Q10 may have a preventive effect on PE. Although, to date, no meta-analyses are available. A RCT that administered 200 mg of coenzyme Q10 (*n* = 118) or placebo (*n* = 117) daily from 20 weeks of gestation until delivery showed a reduced risk of PE (RR: 0.56, 0.33–0.96) [124]. However, the sample size was small, and thus, large-scale RCTs are required. Coenzyme Q10 supplementation is not currently recommended for prevention of PE.

#### Melatonin

Melatonin is a hormone produced primarily by the pineal gland, which regulates the sleep–wake cycle. In addition to its role in sleep, melatonin has multiple functions, including anti-oxidant, anti-inflammatory, anti-aging, neuroprotective, and antihypertensive effects [125–127]. During pregnancy, melatonin is produced in the placenta, resulting in an increase in melatonin levels that are twice as high as those detected in non-pregnant women [128]. In the placenta, melatonin promotes the development of syncytiotrophoblasts, decreases placental oxidative damage, and regulates placental homeostasis [128]. In women with PE, circulating melatonin levels are significantly reduced along with a decrease in synthetic enzymes and melatonin receptors in the placenta [129]. Additionally, based on an in vitro study that demonstrated a reduced secretion of soluble fms-like tyrosine kinase 1, a key factor in PE, from primary trophoblasts following melatonin administration [130], exogenous melatonin supplementation is expected to have potential benefits in PE prevention. However, currently most studies are at the preclinical phase and future clinical studies are warranted to evaluate the preventive effects of melatonin against PE.

#### S-equol

S-equol, a metabolite of soy isoflavones produced by the gut microbiota, is a potent anti-oxidant and has an estrogen-like effect due to its ability to bind selectively to estrogen receptor β [131]. S-equol also has several beneficial effects on vascular endothelial and smooth muscle cells [132]. However, no study has investigated the effects of S-equol supplementation during pregnancy on prevention of PE.

## Conclusion

Among the several dietary supplements evaluated for their preventive effects on PE in this review, calcium is the only supplement with substantial evidence supporting its efficacy. Calcium supplementation has consistently been shown to reduce the incidence of PE, with a daily intake of 500 mg halving the risk of PE. Many Japanese women of reproductive age have an energy intake that is approximately 20% below the recommended level, and up to 45% are deficient in many nutrients, including calcium and vitamin D. Given these circumstances, calcium supplementation during pregnancy is expected to reduce the risk of PE. However, although vitamin D is gaining attention as a potential candidate for PE prevention, the accumulated evidence is not sufficient to recommend routine vitamin D supplementation. Therefore, well-designed, adequately powered studies are needed to obtain additional supporting evidence.
